# Supplementary material for: An inductive bias for slowly changing features in human reinforcement learning
Source: PLoS Comput Biol. 2024 Nov 25;20(11):e1012568. doi: 10.1371/journal.pcbi.1012568 (PMC11637442; doi:10.1371/journal.pcbi.1012568)
Supplement: Attachment — Submitted filename: Response to 1st reviews PLOS CB.docx [file pcbi.1012568.s002.docx]

## Editor:

As you can see all the reviewers found your paper addressing an important question, however the opinion were mixed concerning whether the statistical support to the hypothesis is robust enough (marginal significant test, seemingly skewed distribution of p-values) . Some lack of clarity concerning the relation between the main experiment and pilote and deviations from preregistered analyses also seem to undermine the confidence on the results. On these ground, and in order to allow you presenting a paper that unquestionably support your interesting claims, I would welcome a major-revised manuscript that includes an additional (third) experiment where you assess the (direct) replication of the main findings, thus removing any residual doubt about the statistical and procedural validity of the study. Since the experiments were performed online and the analytical codes are already there, I expect that this should feasible within the delay, but we will of course grant extra time for revising the manuscript, if necessary.

Thank you for these comments. As discussed, and as you will see below, we have performed a third preregistered experiment with n = 138 that largely (although not entirely) confirms our previous results. In our point-by-point reply below we will first repeat each reviewer comment in a box, and then add our reply below. Quoted text from the manuscript is indented and in italics, and new text within these quotes is highlighted in yellow.

## Reviewer #1

Hedrich and colleagues investigate feature learning in a two-dimensional bandit learning task where one of the features changed quickly, while the other changed slowly. The authors report that people pay more attention to features that change slowly, and have a bias in favor of those features even when they are not predictive of rewards. Using formal modeling, the authors found that four separate learning rates are required to properly capture participants' behavior -- paying attention to fast- and slow-changing features in relevant and irrelevant conditions separately.

The good features of the paper include the very good presentation (clear writing, informative and pretty plots) and the interesting topic. I particularly liked the task, it is very clever and well suited to investigate the topic of interest. The only thing that I would change, and I strongly urge the authors to do that, is to remove the choice--information coupling during learning. I do not see any reason why outcome feedback should be coupled to choices, as the main interest lies in feature learning and not in exploration/exploitation. In the present state, there is a strong confound of the potential to learn features and behavioral tendencies such as ambiguity aversion. The models seem decently motivated and well executed, I have few concerns in that aspect.

With all that said, what was submitted as a finished paper serves at most as a registered report. As far as I can tell, the only behavioral result that stands up to rigor is that, on average, participants tended to distinguish good options from bad ones. All other analyses are either misrepresentations of various degrees or simply wrong.

We would like to thank the reviewer for their valuable criticism. In response to your feedback, and in correspondence with the editor, we conducted a direct, preregistered replication of the main experiment in the manuscript.

Briefly, the preregistration targets the 3 main results of the main experiment, the effect of condition on (1) rewards and (2) accuracy in the learning phase and on (3) accuracy in the test phase. Since our hypothesis is clearly directional we preregistered 1-sided tests. The sample size (n = 195, before exclusions) was determined based on power calculations using the test phase effect in our previous experiment (details see below). Preregistered exclusion criteria were determined based on prior results such as to not lead to excessive exclusions and we strictly adhere to them now (effective sample n = 138). The preregistration can be found here: <https://osf.io/v436h?view_only=fe00e38a9b164eaf8ecb1b07c9f8fad1>

It was submitted to OSF on May 15th, prior to starting data collection, and in correspondence with the Editor.

Results show that the condition effects in the learning phase replicate. Participants accumulated more reward in slow compared to fast blocks (M_S_ = 270.2 ± 10.7, M_F_ = 242.9 ± 10.96, t(137) = 2.65, p_1-sided_ = 0.004, d = 0.226). A complimentary logistic mixed effect model confirmed that participant choices reflected the expected rewards more closely in the slow compared to the fast blocks (condition*reward interaction: beta = 0.106, 95% CI = [0.063 to 0.149], likelihood ratio test compared to a model without the interaction term: X^2(1) = 23.32, p < .001). We also confirm the condition difference in choice accuracy (M_S_ = 66.3% ± 0.6, M_F_ = 65.0% ± 0.6, t(137) = 1.99, p_1-sided_ = 0.024, d = 0.169). Additionally, meta-analytic results across the three experiment samples we have collected showed that the learning phase effect is robust (Hedge's g = 0.204, 95% CI = [0.024 to 0.385], Z = 2.22, p = 0.026, heterogeneity: I^2 = 0%, 95% CI = [0%; 90%], Q = 0.01, p = 0.993). Finally, our modelling results remain consistent with our previous results, indicating that the condition effect cannot be attributed to other explanations such as choice repetitions.

The third effect in the test phase did not replicate, i.e. there was no significant difference between conditions in accuracy (M_S_ =74.9% ± 0.9, M_F_ = 74.2% ± 0.8, t(137)= 0.958, p_1-sided_ = 0.170, d = 0.082) or increased effect of reward difference on choices following slow blocks (condition*reward difference interaction: X^2(1) = 2.582, p = 0.216). This replication failure of the test effect is an important nuance to our study and we discuss it appropriately in the manuscript.

Further details can be found in our answers below as well as in the revised manuscript, where all results are reported. Note that in order to help distinguish between samples, we have changed the naming of the pilot task to experiment 1 and main task to experiment 2. Overall, we believe this has made our manuscript significantly stronger and we would like to thank the reviewer for helping us to improve the science through their criticism. An overview of these results is plotted below.

***Fig. R1. Results from the replication. A)****Cumulative reward obtained in a block of the learning phase above a chance baseline of 50 per trial is higher in slow than in fast blocks. Separately for blocks where the slow feature (purple) and fast feature (green) were relevant. Individual participant means in grey.* ***B)*** *Cumulative reward obtained relative to a chance baseline of 50 on each trial increases more rapidly in slow blocks. Grey ribbons show the standard error of the mean.* ***C)*** *Mean accuracy in the test phase is not significantly different in slow and in fast blocks.* ***D)*** *Meta-analysis results for the effect of feature speed on learning phase reward and* ***E)*** *test phase accuracy.* ***F)*** *Protected exceedance probabilities (bars) and estimated frequencies (diamonds) of the models reported in the main paper. Learning models: one learning rate model (1LR), separate learning rates per feature (2LRf ), separate learning rates per condition (2LRc) and the four learning rates model (4LR). Control models: win-stay-lose-shift (WSLS), learning model ignoring features (Bandit), random responding with a bias for accept choices (Rd. Choice) or response key (Rd. Key).*

Finally, we would like to clarify the reasons for the deviations from the previous preregistration. At the time of the first preregistration we did not have data upon which to base the exclusion criterion. Only after data collection did we realise that our exclusion criterion would exclude large parts of the sample, including participants whose behaviour evidently showed they were learning. Rather than choosing another exclusion criterion after already looking at the data, we decided not to use the originally proposed performance-based exclusion criterion for our initial submission. While we believe that we had valid scientific reasons, we understand that this could be considered problematic and are happy that with the new preregistration this concern is now addressed.

**R1C0** (excerpt from the summary): “The only thing that I would change, and I strongly urge the authors to do that, is to remove the choice--information coupling during learning. I do not see any reason why outcome feedback should be coupled to choices, as the main interest lies in feature learning and not in exploration/exploitation. In the present state, there is a strong confound of the potential to learn features and behavioral tendencies such as ambiguity aversion.”

We appreciate and see the merits of the suggestion regarding the experimental design. We first want to note that any changes in design directly conflict with the challenge of a direct replication of our previous results, which the reviewer also suggests. After careful consideration and discussion with the Editor, we decided to focus on the replication, which effectively prevents us from any task adaptations.

This consideration aside, we believe that the explore/exploit dilemma is a core aspect of reinforcement learning, the primary interest of our study. In RL tasks, as in many real-world settings, choosing one option often comes at the cost of foregoing information about other options. Even though the explore/exploit tradeoff is not our main effect of interest, we did not want to test the effect of the slowness prior in a task lacking one of the main aspects of RL.

**R1C1:** First, the preregistration, the data, and analyses. None of these are publicly available, so I could not check what is contained therein and what is not. The authors are transparent about deviations from the preregistration (which is good), but ignoring preregistered exclusion criteria that would remove more than 50% of the sample is bad. The authors claim that "[t]he main results remained unchanged when applying this exclusion criterion" which is plain wrong. In fact, only one of the four results reported after exclusion is statistically significant, and even this one result is probably overly optimistic (see third point below).

We thank the reviewer for these comments and apologise that our data and code were not available to the reviewer. We have uploaded the data and code with our revision to make it available to the reviewers. Both will be made publicly available immediately upon publication.

We hope that our new preregistration (see above) can alleviate any concerns regarding deviations from preregistration and transparency. We are also happy to address the concerns regarding exclusion criteria. As mentioned above, we have defined a new exclusion criterion in the updated preregistration, which we strictly adhere to in our replication. This criterion considers only the last 15 trials of the learning phase and excludes participants whose accuracy lies below the 97.5th percentile of a binomial distribution with probability of success ½. This is the criterion used in the replication study reported in the manuscript.

To determine the sample size we ran a power analysis based on the test phase effect in the previous main experiment (d = 0.26). To achieve ca. 95% power with a paired one-sided t-test we aimed for a sample size of 160 participants (two-sided power ca. 90%). To account for exclusions due to our new data exclusion criterion we collected a total of 195 full datasets, as specified in the preregistration. It turned out that we underestimated the exclusions, which means that only 138 participants passed the exclusion criterion. Since we preregistered it and it is fairly close to the ideal of 160, we worked with this sample size.

We have added a description of the preregistrations to the results as follows:

Lines 150-164

*The three experiments are accompanied by two preregistrations available on OSF (*[*https://osf.io/6dy8f*](https://osf.io/6dy8f)*). The first preregistration was submitted prior to acquisition of any data. We used experiment 1 and 2 to refine the task and analysis. As a result, we deviated from the first preregistration, however note that the hypothesis, core tenets of the task and main analyses are maintained. To validate our results, we updated the preregistration to reflect the changes to the task and analysis and then conducted a direct replication of experiment 2 in strict adherence to this registration. This reregistration focused on 3 main effects: the effect of feature speed on (1) collected rewards and (2) accuracy in the learning phase and on (3) accuracy in the test phase. Three additional analyses that were less central were also included, namely logistic mixed effect models testing the impact of (1) reward and (2) relevant and irrelevant features on learning phase choices, as well as (3) the difference in reward between stimuli on test phase choices. Finally, we included the intent to conduct a meta-analysis across experiments in the registration. In the reregistration we defined a new performance-based exclusion criterion, which led to an effective sample size of 138 participants after exclusions in the replication. The computational models were not included in the preregistrations. For more details on the preregistration see S1 Text.*

We also describe the sample size justification and the exclusion criterion in the methods:

Lines 706-717

*To determine the sample size for the replication study, we ran a power analysis based on the test phase effect in experiment 2, as it was the test with the smallest effect size out of the three main tests (MS = 76%±1.6, MF = 74%±1.5, t(49) = 1.85, p1−sided = .035, d = 0.26). To achieve ca. 95% power with a paired one-sided t-test, to detect an effect with Cohen’s d = 0.26, we aimed for a sample size of 160 participants and collected 195 participants. We introduced a new performance based exclusion criterion in the replication study, to exclude participants with random end-of-learning performance. We defined end-of-learning performance for each participant as the accuracy in the last 15 trials of the learning phase, averaged across all eight task blocks (120 trials). If this mean accuracy was on or below the 97.5th percentile of a binomial distribution over 120 draws with a probability of success of ½ (i.e. 71/120 successes or ca. 59.2% correct), the participant was excluded. After exclusions the effective sample size was 138 participants.*

We also apologise that our statement regarding the effect of the exclusion criterion in the SI was underspecified. This should now be fully addressed with our new experiment. In the original submission our intention was to state that the results stay *qualitatively* the same (see Table R1C1 below for an overview, and note that in one case the result was not significant after exclusion in one instance (at p = .051), but the effect size was consistent, hinting at reduced power causing some differences).

| **Table R1C1.** Overview of the main effect paired t-tests with and without the participant exclusions using the criterion in the first registration. | | |
| --- | --- | --- |
| **Test** | **Without exclusions** | **With exclusions** |
| **Main experiment**  **learning phase reward** | MS = 248.62 ± 21.54,  MF = 217.57 ± 22.43,  t(49) = 2.17, p = .017, d = 0.31 | MS = 351.85 ± 15.53,  MF = 319.13 ± 19.35,  t(29) = 1.69, p =.051, d = 0.31 |
| **Main experiment**  **test phase accuracy** | MS = 76% ± 2,  MF = 74% ± 2,  t(49) = 1.85, p = .035, d = 0.26 | MS = 83% ± 1,  MF = 80% ± 1,  t(29) = 2.08, p = .023, d = 0.38 |
| **Pilot experiment**  **learning phase reward** | MS = 128.11 ± 14.03,  MF = 108.88 ± 14.97,  t(49) = 1.57, p = .061, d = 0.22 | MS = 224.43 ± 15.57,  MF = 214.49 ± 21.30,  t(16) = 0.53, p = .302, d = 0.13 |
| **Pilot experiment**  **test phase accuracy** | MS = 71.3 % ± 2  MF = 71.5 % ± 2  t(49) = 1.14 p = .130, d = 0.16 | MS = 85% ± 2,  MF = 82% ± 2,  t(16) = 1.14, p = .135, d = 0.28 |

**R1C2:** Second, and related, is the overuse of "marginal significance" while operating in the standard NHST framework. I counted at least six such occurrences, and I think this blog post summarizes the problem here the best:<https://mchankins.wordpress.com/2013/04/21/still-not-significant-2/>

We appreciate the reviewer’s dissatisfaction with these statements and have removed them from the paper. We also consistently report the effect sizes alongside the p-values in this and the previous manuscript, which give the reader an insight into the strength of each effect.

**R1C3:** Third, almost all p values of significant effects are just below .05 using one-sided tests. Most of them would disappear using two-sided tests, and especially so with any reasonable correction for multiple comparisons. There are good resources why this is problematic, for example,<https://www.the100.ci/2018/02/15/the-uncanny-mountain-p-values-between-01-and-10-are-still-a-problem/> and<https://quentinandre.net/post/large-pvalues-and-power-analysis/>

As described above, we have conducted a direct replication of the experiment in a larger sample in response to the reviewer’s concerns.

We chose and preregistered one-sided tests because our hypothesis has a clear directionality. Therefore, one-sided tests are a more efficient use of our samples (see: <http://daniellakens.blogspot.com/2016/03/one-sided-tests-efficient-and-underused.html>).

We thank the reviewer for raising the important point on correction for multiple comparisons, we added a section on this to the updated preregistration. We now correct for multiple comparisons in the mixed effect models. We do not correct for multiple comparisons in the t-tests when dependent measures are mathematically related to each other (e.g., higher accuracy in learning must result in higher reward).

Finally, on advice of the editor, we also report meta-analytic measures, the results of which are briefly described above.

**R1C4:** Fourth, the way the two studies are presented is inconsistent with best practices. It seems like not only the analyses, but also the experimental design deviate from the preregistration, which is very odd. Generally speaking, while I would consider reporting a pilot study in the main text and using it to draw inferences acceptable (but not necessarily good practice), I do not understand why the power analysis was conducted using yet another study that is not reported, and why it has not been adjusted after observing the noise in the pilot study. Generally speaking, in line with the first comment on data exclusions, both the pilot and main studies were severely underpowered.

We thank the reviewer for this contribution and apologise that the presentation of our results was not clear.

We hope that our replication of the study in a larger sample will address the reviewers' concerns. For this experiment, we determined the sample size for the replication based on a power analysis of the test phase effect in the main experiment reported in the previous submission (see point R1C1 above).

To clarify, the sample of the pilot experiment (experiment 1) was collected after the preregistration. We named it pilot, to make it intuitively clear that it included less phases than the main experiment (experiment 2) and was collected before the main experiment. We reported this study in the main text, to support our inferences and to acknowledge that we did not find strong evidence for the slowness prior effect in the pilot experiment.

**R1C5:** Fifth, statements about changes in effects are made without supporting them with tests. For example: "however the effect in test phase accuracy was stronger than with the full sample", or "Performance during the test phase did not differ statistically from end-of-learning performance in the learning phase [...]. Hence, our data suggests that participants generalised values successfully across task and stimulus differences between the two phases.". See<http://www.stat.columbia.edu/~gelman/research/published/signif4.pdf> on why these claims are wrong.

We apologise for our underspecified statements, we have removed these statements from the manuscript.

## Reviewer #2

In this paper, the authors test a novel hypothesis of how humans might learn useful priors for task representation, namely whether people are biased to learn more from slowly-changing features in the environment. The experimental task is well designed, the paper clearly written, and the set of questions interesting and under-explored in computational cognitive neuroscience. That said, the conclusions we can draw from the paper, and particularly the computational modeling analysis, could be stronger. Below are some comments that I hope will help sharpen the paper.

**R2C1:** The authors mention running two separate experiments, but it was not clear whether the data shown here is collapsed across the two (e.g. does Figure 2 show data from all 100 participants?); it would be important to know if the presence of an observation phase changes the conclusions of the study.

We apologise that this was unclear and have clearly labelled the plotted samples in all figures (see the example of Fig. R2C1 (Fig. 3 in the paper) below).

Note that, in response to the comments of reviewer 1, we ran a direct replication of the main experiment. For better readability, we have renamed the experiments to experiment 1 (previously pilot), experiment 2 (previously main) and refer to the new sample as the replication. Meta-analytic results across the three samples show that the condition effects in the learning phase replicate (cumulative reward difference: Hedge's g = 0.204, 95% CI = [0.024 to 0.385], Z = 2.22, p = 0.026), but those in the test phase do not (accuracy difference: Hedge's g = 0.080, 95% CI = [-0.100; 0.259], Z = 0.87, p = 0.386). See also Fig. R2C1 (Fig. 3 in the paper), reproduced below.

***Figure R2C1 (Figure 3 paper)****: Participants learned better in slow blocks.* ***a)*** *Cumulative reward obtained in a block of the learning phase above a chance baseline of 50 per trial is higher in slow than in fast blocks in all three samples. Separately for blocks where the slow feature (purple) and fast feature (green) were relevant. Individual participant means in grey.* ***b)*** *Higher cumulative reward in slow compared to fast blocks is confirmed by a meta-analysis across experiments.* ***c)*** *Cumulative reward obtained relative to a chance baseline of 50 on each trial increases more rapidly in slow blocks in all three samples. Grey ribbons show the standard error of the mean.* ***d)*** *Visualisation of the difference in cumulative reward between slow and fast blocks across trials.* ***e)*** *Mean accuracy in the test phase is higher in slow than in fast blocks in experiment 2, but not experiment 1 and the replication.* ***f )*** *Meta-analysis results show that there is no consistent benefit in test phase generalisation for slow blocks.*

In light of this new evidence it seems that the observation phase does not change the conclusions of the study. Rather, it is more likely that the increased trial number in experiment 2 and the replication provides greater power to measure the effect in the learning phase. We have highlighted the difference in trial number more in the results:

Line 134

*The key difference between experiments 1 and 2 was that experiment 2 included a demonstration of stimulus changes before each block. The experiments also differed in the number of trials per block.*

Lines 200 - 208

*In experiment 2 and the replication sample, in line with our hypothesis, the cumulative reward gain was higher in slow compared to fast blocks (preregistered main test 1, experiment 2: MS = 248.62 ± 21.54, MF = 217.57 ± 22.43, t(49) = 2.17, p1−sided = .017, d = 0.31, N slow>fast = 33/50; replication: MS = 270.185 ± 10.730, MF = 242.95 ± 10.96, t(137) = 2.654, p1−sided = .004, d = 0.23, N slow>fast = 86/138; Fig 3a,c). In experiment 1, in which the observation phase was omitted and blocks were shorter, participants accumulated higher reward on slow blocks, but the difference was not significant (MS = 128.11±14.03,MF = 108.88 ± 14.97, t(49) = 1.57, p1−sided = .061, d = 0.22, N slow>fast = 30/50, Fig 3a,c).*

We also included trial numbers in Figure 1:

**R2C2:** Upon further reading, the pilot experiment was analyzed separately and included in the supplement: please make this clear in the subheadings earlier in the paper, and by adding the sample size to the plots in Figure 2.

We thank the reviewer for this suggestion and apologise that it was unclear which experiment was being reported. In all figures it is now clear which sample size is being plotted (see the example of Fig. R2C1 (Fig. 3 in the paper) above). We now report the samples from all three studies in the main text to make the comparison between them easier. Therefore, we did not change the subheadings.

**R2C3:** Did the performance difference between conditions emerge gradually? That can be addressed by plotting 2a as a function of condition and including trial as a variable. We would expect that to be the case based on 2f, since correct acceptance should increase cumulative reward.

The reviewer makes an interesting point. The plot in Figure 2a split by condition is shown below (Fig R2C3 A). Additionally, we plotted the difference in the proportion of correct responses for each condition across trials (Fig R2C3 B). The performance difference seems to emerge within the first ten to fifteen trials and remain relatively constant throughout the block.

To test this, we compared mean participant accuracy in slow vs. fast blocks in bins of 15 trials, pooling data from all three experiments (N=238). Higher accuracy in slow compared to fast blocks was first found in the second bin (trials 16-30) and was also visible in the bins thereafter, though one test did not survive correction for multiple comparisons (trials 31-45 & 46-60). In the first bin (trials 1-15) we found no significant difference between conditions. See table R2C3 below for the statistics.

We have added the learning curve split by condition to the supporting information. As pointed out by the reviewer, the performance plot shows a picture consistent with the cumulative reward plot (Fig. 2f in the original submission, Fig. 3b in the current submission), however the cumulative reward is a more powerful measure of performance.

| **Table R2C3.** Table showing the two-sided t-tests comparing participant choice accuracy in the learning phase in bins of 15 trials. Pools data from all three experiments. | |
| --- | --- |
| **Trials** | **T-Test (all p-values corrected for multiple comparisons)** |
| 1-15 | M_S_ = 53.2% ± 0.3, M_F_ = 52.7% ± 0.4, t(237) =1.004, p =.316 |
| 16-30 | M_S_ = 64.1% ± 0.7, M_F_ = 62.5% ± 0.6, t(237) =2.646, p =.035 |
| 31-45 | M_S_ = 70.1% ± 0.7, M_F_ = 68.6% ± 0.8 , t(237) =2.214, p =.056 |
| 46-60 | M_S_ = 75.0% ± 0.8, M_F_ = 73.1% ± 0.8, t(237) = 2.554, p =.035 |

***Fig R2C3. A)*** *Accuracy in the slow and fast condition, averaged in a 3-trial sliding window.* ***B)*** *difference in accuracy between slow and fast conditions across trials, black line: averaged in a 3-trial sliding window, blue line: smoothed using locally estimated scatterplot smoothing (LOESS) as implemented in R ggplot2.*

**R2C4:** If we zoom in on Figure 2F, first 10 trials, it appears that the pattern of results is flipped, such that there is an initial decrease in cumulative reward for the "slow" condition. Seeing as fast-changing features are initially more likely to capture attention (<https://jov.arvojournals.org/article.aspx?articleid=2750470>), it would be interesting to understand how this effect interacts with the slow feature bias.

Thank you for bringing this interesting idea to our attention. As pointed out, the cumulative reward in the slow condition appears to decrease in the first 10 trials (Fig R2C4 A and zoomed in B).

We quantified the individual extent of this decrease in experiment 2 as the mean cumulative reward relative to chance in the first 10 trials in each block, in each condition (Fig R2C4 C). This confirmed a lower mean cumulative reward relative to chance in the first 10 trials for the slow condition. However this difference was not significant and there was high variability between participants, indicating this might be a spurious effect (slow: M=-8.25, SD=36.5, fast: M=-4.19, SD=20.2, t(49) = -0.687, p = .495).

We asked whether this dip in reward has any relation to our main effect by correlating it to the difference in performance between slow and fast blocks in experiment 2. We did not find a significant correlation with the difference in cumulative reward in the learning phase (r = .11, p-value = .444, D) or with the difference in accuracy in the test phase (r = .12, p = .397, E). We also did not find a correlation between the *difference* in reward in the first ten trials between slow and fast conditions and the cumulative reward in the learning phase (r = .13, p = .368 F) or difference in accuracy in the test phase (r = .14, p = .339 G). Hence, We did not find a relationship between this initial dip in reward and the effect of the slow feature bias in our data.

***Fig R2C4. A****) Figure 2F as in the initial submission* ***B****) the same plot as in A, zoomed in on the first 15 trials* ***C****) mean cumulative reward relative to chance in the first 10 trials of each block for the slow and fast conditions.*

**R2C5**: The lack of a condition difference in the pilot experiment suggests a top-down mechanism by which people select which features to pay attention to. What could this mechanism be? One can imagine different explanations (e.g. reducing visual working memory load), it would be worthwhile to consider some in the discussion.

We thank the reviewer for this proposal. As described above, the new evidence from the replication study and meta-analysis across studies indicates that the lack of a condition effect in experiment 1 (pilot) might be due to the lower number of trials in this experiment, rather than due to the lack of the observation phase. Nonetheless, the reviewer raises an interesting point, which we have expanded upon in the discussion:

Lines 447 - 450

*Our work relates to a broader discussion of how the human brain solves representation learning problems [27, 30]. Previous work has shown how representation learning can be implemented in parallel to reinforcement learning by using feedback signals to guide selective attention [57, 58], or through replay mechanisms during offline periods [59, 60]. Although these approaches represent flexible mechanisms that allow on-the-fly adaptation to the current environment, it is unlikely to be feasible in environments with hundreds of possible signals to attend to [3, 6, 29]. Our results suggest that for this reason representation learning mechanisms during RL are supplemented with inductive biases. Inductive biases may act by biasing beliefs about which features are relevant, thereby reducing the reliance on online computation. This narrowing down of options could be implemented as selective attention, which recruits frontal, parietal and visual cortical areas [61]. One possibility is that inductive biases modulate activity in these areas to accelerate representation learning. Our findings are in line with previous research showing that priors have a pervasive influence on behaviour, shaping perception [15, 37], remaining stable in the face of exposure to contradictory training [62], and hindering learning of structures which do not align with them [63, 64]. [...]*

**R2C6**: I was a bit confused by the interpretation of the computational models. As I understand it, the winning model allows for LR differences based on condition (slow vs. fast). That seems reasonable, as people already know which feature changes slowly vs. quickly from the observation phase. However, allowing the model to also know the relevant feature in advance is peeking, since people have to learn it from trial and error. So the 2LR_c (relevant feature changing fast and slow) model and 4LR model (both relevant and irrelevant feature changing fast and slow) seem like oracle models that, unsurprisingly given number of trials, likely recapitulate the (asymptotic?) learning effect without modeling it directly. Can the authors clarify the choice of modeling approach?

The reviewer is correct that any model which assumes different learning rates between relevant and irrelevant features from the onset has an “oracle” element to it. While it should be noted that our function approximation approach does in fact model learning of feature relevance through the model weights, we agree that our model assumes a bias in this learning process that anticipates the outcome of it.

The fact that a model with this assumption is the best fitting model (the 4LR model, see Fig R2C6 below) indicates that participants infer feature relevance very quickly in our task. This effectively minimises the difference between our model and a model in which inference of feature relevance would gate learning rates. Our early attempts to devise a model wherein the learning rate is coupled to the inferred feature relevance proved difficult to reconcile with the learning rate updates necessary for the Kalman Filter aspect of the models (for reasons why the Kalman Filter aspect is necessary see R3C4 below). As these models were ill behaved, we did not pursue them further.

***Fig. R2C6****. Model comparison using protected exceedance probabilities based on AICc weights.* ***A)*** *experiment 2* ***B)*** *replication. These plots are included in the supplementary information Figure S4. Note that the model comparison results for experiment 2 are slightly different as we corrected a mistake in the sample size used for the AICc calculation. Previously we wrongly used 50 and now have corrected this to the number of trials used for obtaining the sum log likelihood of the model (480 - no response trials).*

We recognize that this is a shortcoming of our work that future studies should address and therefore have included the following section in the discussion:

Lines 403 - 500

*Our task and models make some simplifying assumptions. [...] Our winning model, the four learning rate model, assigned learning rates to the features based on their speed and relevance from the first learning trial of the block. While it is reasonable to assume that participants in experiment 2 and the replication knew the speed of the features based on the preceding observation phase, they could not yet have known which feature was relevant. The models gradually learn the feature relevance through the feature weights, but the structure of the learning rates anticipates the outcome of this learning process. Nonetheless, the fact that a model with this assumption fits best and participants' accuracy increased within the first learning trials in a block, lead us to believe that participants quickly developed a sense for the relevance of the features. We chose this approach for its computational simplicity, but it remains a potential avenue for future research to set up models which do not assign relevant and irrelevant learning rates a priori, but infer these assignments through learning. [...]*

**R2C7**: More broadly, the interesting question the authors set out to answer is, do people have a bias to learn from slowly-changing features first? The paper provides evidence for this in the form of a behavioral effect, but this conclusion is less warranted by the computational analysis. To show that, you would need a model that explicitly infers feature relevance and biases that inference, or at least early exploration, in favor of slow-changing features.

This is an interesting and important point. Our function approximation models infer relevance indirectly through feature weights, which are initially set to a baseline value and updated following each choice. Feature relevance then emerges in the form of the absolute magnitude of the weights – features with large weights affect the value expectation greatly, while features with weights close to baseline are effectively ignored. In this process, the learning rate is a decisive factor. A learning rate of 0 for some features would make it impossible to ever learn that this feature is relevant. A learning rate of 1 would imply that it will never be possible for the model to ignore this feature, even when it is irrelevant.

The core aspect of our model is that it assigns a different (higher) learning rate for slow than for fast features a-priori. This higher learning rate therefore leads to faster learning when the slow feature is relevant, and slower learning of relevance when the fast feature is relevant, effectively implementing our hypothesis.

To illustrate this, below we plot the average absolute weights of our fitted 4LR model for slow versus fast blocks over time. As can be seen, the fitted model infers feature relevance faster in the former compared to the latter case (Fig. R2C7).

***Figure R2C7****.* ***A)*** *Sum of the average absolute weights of our fitted 4LR model for slow versus fast blocks over time for the sample of experiment 2. Averaged over both feature dimensions.*

The winning model also captures the core aspects of behaviour well, both qualitatively as well as quantitatively, including the behavioural effect the reviewer alludes to. Hence, we believe that our model does add significantly to answer the question at the heart of our manuscript. We apologise that this has not become clear in our previous version. To correct this issue, we have now inserted the following explanation in the text:

Lines 318 - 321

*While all of the four models reported here used the above-described mechanisms, they differed in whether they could adapt their learning rates to the slowness of the features, the relevance of the features to predict reward, or both (see Fig 4 right column). A baseline model used the same learning rate α for all conditions and features (one learning rate model, short 1LR). Hence, this model was indifferent to slowness and could not account for a difference in performance between the slow and fast blocks. A second model used separate learning rates for the slow vs. fast-changing feature (αS /αF ), irrespective of whether the feature was relevant in a given block (feature learning rates model, 2LRf ). This model could account for the difference in performance between slow and fast blocks, but since it disregarded the relevance of the features for predicting reward it is an unlikely candidate to explain participant behaviour a priori. In a third model (condition learning rates model, 2LRc), separate learning rates were used depending on whether the relevant feature was changing slowly (αS ) or quickly (αF ), but used the same learning rate for both features within the same block, regardless of their relevance. Finally, the fourth model had four separate learning rates for the slow and fast-changing features, when they were relevant and irrelevant (4LR model, learning rates αS,R, αF,R vs αS,I , αF,I , respectively). This model could accommodate both differences in learning due to the slowness of the features and the reward structure of the task, for which reason we expected this model to predict participant choices best. The core aspect of these models is that they can assign different learning rates to the slow and the fast features or blocks a-priori. Should the learning rate for the slow feature or condition be higher, this would lead to faster learning when the slow feature is relevant, and slower learning when the fast feature is relevant, effectively implementing our hypothesis.*

**R2 Minor:** Figure 3: please explain acronyms (e.g. 2LRc and 2LRf) in the figure caption

Thank you for bringing this to our attention. We added the following text to the Figure 3 caption:

*[…] The right column shows how the learning rates map onto the stimulus features and experimental condition. Learning models: one learning rate model (1LR), separate learning rates per slow/fast feature (2LRf ), separate learning rates per slow/fast condition (2LRc) and the four learning rates model (4LR).*

## Reviewer #3

Thank you for the opportunity to review this manuscript entitled, “An inductive bias for slowly changing features in human reinforcement learning.” In this paper, the authors tested the hypothesis that humans learn better from reward-predictive features when those features change slowly over time. Moreover, this learning bias is assumed to result from people’s prior knowledge that, in most environments, signal tends to change on a slower timescale than noise. Participants completed a bandit task in which the options had two features (color/shape), one changing slowly and the other changing quickly within each block of trials. Moreover, one of the features (slow or fast) was predictive of reward, while the other was not. Participants obtained greater cumulative reward and achieved better generalization performance when the reward-predictive feature changed slowly and the irrelevant feature changed quickly. Reinforcement learning (RL) models suggested that learning rates were higher for slow features, but only when those features were relevant to the task (i.e., predictive of reward).

Overall, I thought the manuscript was well-written, the methods appropriately chosen, and the analyses carefully and correctly executed. I think the research question is interesting and the results were generally supportive of the hypotheses. I have a few questions, comments, and suggestions that I hope will be constructive to the authors.

We thank the reviewer for their helpful comments. To preface our responses we wanted to note that we ran a direct replication of the main experiment in response to the comments of reviewer 1. Briefly, results across the three samples show that the condition effects in the learning phase replicate (meta-analysis across the three experiments’ cumulative reward condition difference: Hedge's g = 0.204, 95% CI = [0.024 to 0.385], Z = 2.22, p = 0.026), but those in the test phase do not (accuracy condition difference: Hedge's g = 0.080, 95% CI = [-0.100; 0.259], Z = 0.87, p = 0.386). See also Fig. R3C0 (Fig. 3 in the paper), reproduced below. For better readability, we have renamed the experiments to experiment 1 (previously pilot), experiment 2 (previously main) and refer to the new sample as the replication.

***Figure R3C0 (Figure 3 paper)****: Participants learned better in slow blocks.* ***a)*** *Cumulative reward obtained in a block of the learning phase above a chance baseline of 50 per trial is higher in slow than in fast blocks in all three samples. Separately for blocks where the slow feature (purple) and fast feature (green) were relevant. Individual participant means in grey.* ***b)*** *Higher cumulative reward in slow compared to fast blocks is confirmed by a meta-analysis across experiments.* ***c)*** *Cumulative reward obtained relative to a chance baseline of 50 on each trial increases more rapidly in slow blocks in all three samples. Grey ribbons show the standard error of the mean.* ***d)*** *Visualisation of the difference in cumulative reward between slow and fast blocks across trials.* ***e)*** *Mean accuracy in the test phase is higher in slow than in fast blocks in experiment 2, but not experiment 1 and the replication.* ***f )*** *Meta-analysis results show that there is no consistent benefit in test phase generalisation for slow blocks.*

**R3C1**: As I was reading pp. 2-3, the immediate question I had was whether people are actually biased toward focusing on slowly changing features, or if it is simply easier to detect/predict them? I would appreciate if the authors could discuss in greater detail whether their results can help answer this question.

We thank the reviewer for raising this interesting point.

First, we would like to argue that the two possibilities, being biased toward focusing on slow features or it being easier to predict slow features, are not mutually exclusive. If one considers that a goal of representation learning is to distinguish signal from noise and that signal is more predictable than noise, it follows that one should pay more attention to more predictable features. It is possible that people pay more attention to features, *because* they are more predictable.

In our experiment we do not directly measure attention, so we cannot conclusively answer this question. We do test whether participants exploit the trial-to-trial predictability of the slow features (e.g. is the feature/outcome I just saw likely to be repeated on the next trial) by fitting a win-stay-loose-shift (WSLS) model. This model did not explain participant behaviour well, indicating that this simplified strategy which could facilitate learning does not account for our results.

We have updated the introduction and discussion accordingly:

Introduction lines 81 - 83

*A useful prior for reinforcement learning should therefore help quickly build appropriate task states from rich perceptual observations in novel environments [8, 30]. A characteristic shared across many environments is that the causal process generating observations develops on a slower timescale than the sensory signals we observe [31–33]. For example, the appearance of a ball flying toward you in a park might fluctuate rapidly as it passes through patches of sun and shade, but its true colour will remain unchanged. Similarly, other relevant properties such as its speed and trajectory will change in a slower, continuous manner compared to low-level perceptual features. In short, signal tends to vary more slowly than noise [34]. Consequently, slow features are both more predictable, making them easier to learn from, and useful for predicting future observations, therefore warranting greater attention [35, 36]. It follows that a way to extract features relevant to building task states, while remaining impartial to the exact nature of those features or the causal process underlying the perceptual observations, is to focus on slowly changing features. Indeed, some research has shown that humans have a bias toward perceiving slower speeds in the spatial domain [34, 37, 38]. This idea has gained more traction in machine learning, where a slowness prior has been shown to enable the discovery of task states from raw observations [8, 28, 39, 40]*

*Discussion lines 458 - 460*

*While our results align with several theoretical studies on the slowness prior [34, 39, 43], it is important to consider other ways in which slowness can benefit learning. By their nature, slow features are more predictable [35, 36]. This raises the question of whether the learning improvement for slow features is due to them being easier to learn from, rather than being selectively attended to. For instance, the temporal auto-correlation of features and rewards inherent to a slowly changing461*

*environment could enable the use of heuristic strategies, such as a win-stay-lose-shift rule [49, 50]. We addressed this concern through model comparison and found that these strategies were unable to explain the behaviour of participants. Another possibility is that presenting stimuli in an ordered fashion yields benefits, as suggested in function learning studies [66]. In our task, slow blocks were more likely to be ordered than fast blocks, but due to the periodic nature of our feature-reward mapping, ordering might not be immediately apparent in either condition. Still, future research should aim to disentangle the effects of ordering and slowness on learning. Importantly, assuming relevant processes change slowly only is a useful assumption given the physical laws that govern our world, i.e., Newton’s first law of motion, inertia [39]. Under these conditions, slow acceleration and changes in acceleration are likely to also provide useful priors, as has been shown in motion perception studies in humans [38]. Human learning likely incorporates a host of priors, reflecting other properties determined by our (intuitive) physical understanding of the world [16].*

**R3C2**: I suggest reporting somewhere in the main text the number of individual participants who showed significantly better learning in slow compared to fast blocks (i.e., in both cumulative reward earned and generalization performance).

Thank you for this idea, we have added the numbers of participants showing the predicted effect whenever reporting one of the three main t-tests. See below the example of the test for the cumulative reward in the learning phase:

Lines 200 - 208 (underline added)

*In experiment 2 and the replication sample, in line with our hypothesis, the cumulative reward gain was higher in slow compared to fast blocks (preregistered main test 1, experiment 2: MS = 248.62 ± 21.54, MF = 217.57 ± 22.43, t(49) = 2.17, p1−sided = .017, d = 0.31, N slow>fast = 33/50; replication: MS = 270.185 ± 10.730, MF = 242.95 ± 10.96, t(137) = 2.654, p1−sided = .004, d = 0.23, N slow>fast = 86/138; Fig 3a,c). In experiment 1, in which the observation phase was omitted and blocks were shorter, participants accumulated higher reward on slow blocks, but the difference was not significant (MS = 128.11±14.03, MF = 108.88 ± 14.97, t(49) = 1.57, p1−sided = .061, d = 0.22, N slow>fast = 30/50, Fig 3a,c).*

**R3C3**: The computational modeling analysis assumes that the effects of slow vs. fast changing features are mediated by the learning rate parameter. This seems plausible, but have the authors tested alternative possibilities? For example, could the results be explained equally well if a different parameter (e.g., the noise parameter, sigma^2) varied across conditions?

Thank you for this suggestion. In response, we added two models that allow other parameters to vary by condition. The first varies the exploration parameter **c**, which mediates the extent to which uncertainty increases the value of accepting (1LR_condC). The second varies the decision noise **sigma** by condition (1LR_condSD). Otherwise, both are identical to the one learning rate model (1LR). The figure **R3C3** below shows the results of the model comparison when including these additional models. The 1LR_condSD model does explain the data of some participants, however it does not outperform the two best models (4LR and 2LR_C). As these results do not change the conclusions of our results, we have added these analyses to the supporting information.

We describe these models in the methods:

Lines 937 - 943

*To test whether parameters other than the learning rate could explain differences in participant behaviour between the slow and fast blocks, we fit two additional learning models. The first model allowed the the exploration parameter c, which mediates the extent to which uncertainty increases the value of accepting (see equation 12), to be different for slow and fast blocks (1LRc). The second model allowed the decision noise parameter σ2 to vary by condition (1LRσ2 , see equation 14). Otherwise, both were identical to the one learning rate model (1LR). The results of these models are only included in the supplementary information in S4 Fig.*

and allude to the results of these models in the main text:

Lines 367 - 369

*We fit additional models which allowed parameters other than the learning rate to vary by condition, however these provided a worse account of participant behaviour and are not included in the analyses above (see S4 Fig).*

***Fig. R3C3.*** *Model comparison using protected exceedance probabilities based on AICc weights.* ***A)*** *experiment 2* ***B)*** *replication. These plots are included in the supplementary information Figure S4. Note that the model comparison results for experiment 2 are slightly different as we corrected a mistake in the sample size used for the AICc calculation. Previously we wrongly used 50 and now have corrected this to the number of trials used for obtaining the sum log likelihood of the model (480 - no response trials).*

**R3C4**: In the Kalman filter-based models considered here, learning rates decrease across trials. How dependent are the conclusions on this particular choice of model? Would the results hold if learning rates were constant across trials?

Thank you for this relevant question.

We chose to use Kalman-filter based models, as they provide a principled estimate of uncertainty that we could use as an exploration bonus. This exploration bonus proved to be essential in fitting participant behaviour well, due to the strong tendency in participants to explore, particularly at the start of the block. We initially fit models with fixed learning rates, where we accounted for exploration by including additional parameters that could increase (or decrease) the value of an ‘accept’ choice. We plotted one of these models below, which uses a single fixed learning rate and ‘accept’ bias (1LR_fixedLR).

As can be seen by the mean AICc (Fig. R3C4 A) and proportion of ‘accept’ choices over trials (Fig. R3C4 B), this model family did not account well for participant choices. Particularly, it failed to capture the exploration period early in the learning phase. For this reason, we did not pursue these models further and replaced them with the Kalman-filter based models.

***Fig. R3C4.*** ***A)*** *Difference in mean AICc between the 1LR model and the remaining models. A smaller value means better fit.* ***B)*** *Mean proportion of ‘accept’ choices over trials in the blocks for participants (black) and the 1LR_fixedLR model (yellow). Grey ribbon shows the standard error of the mean. Both plots show the sample of experiment 2.*

**R3C5**: Line 195: should this be Fig. 2f?
**R3C6**: Line 217: should this be Fig. 2g?
**R3C7**:. Line 839: should be either maximizing LL or minimizing negative LL.

Thank you for pointing this out. We corrected all three instances in the text.
